# Education and WHO Recommendations for Fruit and Vegetable Intake Are Associated with Better Cognitive Function in a Disadvantaged Brazilian Elderly Population: A Population-Based Cross-Sectional Study

**DOI:** 10.1371/journal.pone.0094042

**Published:** 2014-04-15

**Authors:** Maria Pastor-Valero, Renata Furlan-Viebig, Paulo Rossi Menezes, Simon Almeida da Silva, Homero Vallada, Marcia Scazufca

**Affiliations:** 1 Departamento de Salud Pública Historia de la Ciencia y Ginecología, Universidad Miguel Hernández, España; 2 CIBER en Epidemiología y Salud Pública (CIBERESP), Madrid, España; 3 University of São Paulo, Faculty of Medicine, Department of Preventive Medicine, Brazil; 4 University of São Paulo, Faculty of Medicine, Institute of Psychiatry and LIM-23, Brazil; Oregon Health & Science University, United States of America

## Abstract

Brazil has one of the fastest aging populations in the world and the incidence of cognitive impairment in the elderly is expected to increase exponentially. We examined the association between cognitive impairment and fruit and vegetable intake and associated factors in a low-income elderly population. A cross-sectional population-based study was carried out with 1849 individuals aged 65 or over living in São Paulo, Brazil. Cognitive function was assessed using the Community Screening Instrument for Dementia (CSI-D). Fruit and vegetable intake was assessed with a Food Frequency Questionnaire (FFQ) and categorized into quartiles of intake and into total daily fruit and vegetable intake using the cut-off points for the WHO recommendations (<400grams/day or ≥400 grams/day). The association between cognitive impairment and each quartile of intake, and WHO recommendation levels, was evaluated in two separate multivariate logistic models. The WHO recommendations for daily intakes ≥400 grams/day were significantly associated with 47% decreased prevalence of cognitive impairment. An effect modification was found in both models between cognitive impairment and “years of education and physical activity” and “years of education and blood levels of HDL” So that, having 1 or more years of education and being physically active or having 1 or more years of education and levels higher than 50 mg/dl of HDL-cholesterol strongly decreased the prevalence of cognitive impairment. In this socially deprived population with very low levels of education and physical activity and fruit and vegetable intake, those who attained WHO recommendations, had 1 year or more of education and were physically active had a significantly lower prevalence of cognitive impairment. A more comprehensive understanding of the social determinants of mental health is needed to develop effective public policies in developing countries.

## Introduction

Due to increased longevity worldwide, cognitive decline and dementia are increasing exponentially [1]. Increasing evidence suggests that aging and age-dependent accumulation of mtDN [2] lead to the production of free radical species (ROS), resulting in mitochondrial and synaptic damage in neurons which is present from the early stages of Alzheimer disease (AD) [3]. Antioxidant nutrients, which can scavenge free radicals, might have the potential to delay cognitive decline and prevent progression to dementia [4]. In fact, research focusing on developing mitochondria-targeted antioxidants appear to be promising for the treatment of AD [3].

Fruit and vegetables are rich in antioxidant micronutrients which have anti-inflammatory properties that may offer neuroprotection [5]. In 2004, the WHO launched a strategy to reduce the prevalence of non-communicable diseases. This included eating 5 or more portions of fruit and/or vegetables a day (≥400 g/day) [6]. Despite the fact that some studies have examined the relation between fruit and vegetable intake and cognitive function, no studies have examined whether these recommendations may offer protection against cognitive impairment. A recent systematic review included a total of nine studies [7]. Six analysed fruit and vegetable intake separately. Of these, five found a positive association with vegetable consumption and decreased risk of cognitive function or dementia, but not with fruit intake, one did not find an association with either fruit or vegetables, and three studies found a positive association between combined intake of fruit and vegetables and decreased risk of cognitive impairment or dementia.

Brazil has one of the fastest-aging populations in the world, with prevalences of dementia ranging from 5,1% to 8,8%. However, few studies have been carried out to assess cognitive impairment [1], [8]–[10], and only one looked at the association between fruit and vegetable intake and cognitive function [11].

Other factors such as sociodemographic characteristics, health behaviours, genetic factors and chronic diseases have been associated with cognitive decline and dementia [12]. Education may also be an important factor. It shapes food choices [13], but may also contribute to cognitive reserve (CR). Studies suggest that, although education is not directly related to the neuropathologic lesions, it appears to reduce the impact of such lesions by increasing the cognitive reserve. However, most of these studies have been carried out in populations with high levels of education (9–18 years).

Physical activity has also been suggested as having a protective effect against cognitive decline [14], [15], although other studies have concluded that there is little evidence to establish such an association [16].

This study aimed to examine the relationship between cognitive impairment and daily intake of fruit and vegetables, including ¨five a daÿ WHO recommendations, in an elderly disadvantaged population of São Paulo city. We also assess the relationship between cognitive impairment and factors such as years of education, APOE gene, co-morbidities and lifestyle variables.

## Materials and Methods

### Study Design

A cross-sectional population-based study was carried out during 2003–2005 with elderly participants of the *São Paulo Ageing & Health Study* (SPAH). The SPAH aimed at estimating the prevalence of dementia, cognitive decline and associated factors including consumption of fruit and vegetables from residents aged 65 years or over living in an economically deprived area of São Paulo city, Brazil. The present study used the protocol developed by the 10/66 Dementia Research Group, an international network of investigators with the principal objective of estimating the prevalence of dementia and risk factors in elderly populations in low- and middle-income countries [17].

A detailed description of the protocol as well as the methodology of the SPAH study has been published previously [9].

### Study Population

Briefly, according to the most recent 2010 census, São Paulo city has an estimated population of 11.2 million, including over one million people aged 60 or over [18]. The city is divided into 31 administrative boroughs. The SPAH study aimed to enrol all residents ≥65 years of age, in 66 census sectors, belonging to three Butantã boroughs in the western region of the city. The study covered a population of approximately 63,000 residents, representing 17% of the total population of these boroughs. The census sectors included were characterized mainly by the presence of shanty towns and/or Family Health Program teams. These areas had the lowest Human Development Indexes of the 3 boroughs [19]. Eligible participants were residents of the defined census sectors. They were identified and recruited through door-knocking of all households within the census sectors boundaries. All eligible subjects were invited to participate. Institutionalized individuals were not included. A total of 2000 individuals were required to detect a prevalence of dementia of 5.0% with 80% power, 5% significance and 95% confidence interval ranging from 4% to 6%. For that, it was necessary to contact 21,727 residences from the 66 census sectors to identify 2266 potential participants. From these, 2072 agree to participate, thus response rate was 91.4%. The population of the present study was selected from the participants of the SPAH study, excluding those with a dementia diagnosis at entry (n = 105). Ethical approval no. 361/07 was obtained by the Local Ethical Committee of Diretoria Clínica do Hospital das Clínicas e da Faculdade de Medicina da Universidade de São Paulo. Written informed consent was obtained from all subjects, and for those with cognitive impairment consent was obtained from the informants.

### Data Collection

#### Procedures

Eight mental health workers were trained in the use of the research protocol including the standardized questionnaires about socioeconomic status and medical characteristics. For each participant, an informant was also identified. Informants were co-residents aged 16 years or over, or a relative or friend who was familiar with the participant’s life history. For participants with severe mental or physical disabilities, informants were asked about the participant’s socioeconomic status and medical characteristics. Only 1.6% (n = 29) of the interviews were answered by the informants (informants’ mean age was 50), with 75.9% of the interviews (n = 22) from participants with cognitive impairment.

Interviews with participants and informants were scheduled to take place at the participant’s home approximately one week after recruitment. These assessments took approximately 90 minutes. A nursing assistant conducted the measurement of blood pressure and collected blood samples at the participant’s home, 2–15 days after the assessment interview.

#### Measurement of cognitive function and cognitive impairment

The assessment of cognitive function was carried out using the protocol developed by the 10/66 Dementia Research Group for use in population-based studies in developing countries and validated in elderly Brazilians from three different cities including São Paulo [17], [20]. The Community Screening Instrument for Dementia was used to assess cognitive function (CSI-D) [21]. This instrument was devised to avoid educational and cultural bias including illiteracy and low levels of education. It consisted of 32 items which assessed cognitive abilities (memory, abstract thinking), language (aphasia, agnosia and semantic verbal fluency), praxis (motor response) and orientation (spatial and temporal). In this study, cognitive impairment was defined as any score from −1.5 standard deviations from the population cognitive function mean score (mean = 26.93, SD = 3.98) [22].

#### Consumption of fruit and vegetables

Fruit and vegetable intake of each participant was obtained from the participants’ responses to the fruit and vegetables section of a Food Frequency Questionnaire (FFQ). Further details of the design of this FFQ were published previously [23]. Briefly, this FFQ, a Harvard-liked questionnaire, was developed to obtain information on the diet of the general adult population residing in the Metropolitan Region of São Paulo. The FFQ had nine possible response categories for frequency of consumption of each item, ranging from never or less than once in a month to 6 or more times per day. The FFQ assessed how often each one of the food items had been consumed by the elderly in the last 12 months. The vegetables group included ten items, and the fruit and natural juices included 17 food items. The number of daily servings of fruit and vegetables per individual was obtained from the sum of all the FFQ responses referred to the mentioned food items and converted into grams/day of total fruit and vegetable intake. Characteristics of the consumption of fruit and vegetables by this population using this FFQ have been previously published [24].

#### Co-variates and confounding variables

Information on co-variates was collected using structure questionnaires for socio-demographic aspects (age, sex, marital status, years of education: none, 1 to 3 years, 4 or more, and per capita monthly income: in minimum Brazilian Real salaries at the time of the study); lifestyle variables including self-reported physical activity which was collected by asking: ¨taking into account both working and leisure time would you consider yourself: sedentary, moderately active, or highly activë. Information on ever having smoked (yes/no), usual alcohol consumption (yes/no), monthly consumption of fish (never, some days, frequently), self-reported history of diabetes, hypertension, and previous cerebrum-vascular accident, was also collected. Increasing evidence indicates a potential relation between obesity and cognitive deficits [25]. Thus, nutritional status was assessed calculating Body Mass Index (BMI) using the weight in kilograms divided by the square of the height in meters. Height was measured to the nearest 0.1 cm with subjects standing without shoes. The BMI was classified according to the WHO cut-off points for elderly population [26]. Blood was collected for analyses of fasting glucose (mg/dL), total cholesterol (mg/dL), HDL-cholesterol (mg/dL), LDL-cholesterol (mg/dL), VLDL-cholesterol (mg/dl) and triglycerides (mg/dL). HDL-cholesterol was re-categorized according to the American Heart Association guidelines [27], [28]. Thus, we redefined HDL levels into two categories low levels ≤50 mg/dl were considered to be associated to risk of cardiovascular disease and high levels >50 mg/dL were considered to be protective against cardiovascular risk. Triglycerides were not considered into multivariate analyses since there was a high number of missing values (65.5%). APOE gene was analysed and individuals carriers of two e4 alleles were categorized into the high risk group APOE = e4/e4, those with one e4 allele were considered to have a moderate risk APOE = e4/e3, e4/e2, those with e2/e2 were considered to have a protective genotype, whereas carriers of e2/e3 were chosen as the reference group and classified as having neither risk nor protection. We found just one individual with APOE e4/e4 and cognitive impairment, so that we had to collapse the two risk categories into one risk category: any individual carrier of e4/e4 or e4/e3, e4/e2 for further statistical analysis. Mean values of systolic blood pressure (mmHg) and diastolic blood pressure (mmHg) were obtained from measurements taken in triplicate.

### Statistical Methods

The cognitive function was built from scores obtained using the CSI-D instrument resulting into a continuous variable whose values could range between 1 to 34 points, the lower the score the greater the cognition decline. The CSI-D scores were not normally distributed. Thus, the multivariate linear models did not meet the assumptions of normality and homoscedasticity despite the different transformations used in order to reach these assumptions. Therefore, the multivariate linear models were finally not used in our analyses. We define cognitive impairment from the continuous variable, cognitive function, as a dichotomous variable (yes/no), based on cognitive scores less than or equal to 1.5 standard deviations below the mean score of the population under study: ≤20, 9 score. Main exposure, total daily combined intake of fruit and vegetables, was converted into grams per day for each participant. This final continuous variable was first re-categorized into quartiles of intake and also re-categorized using the cut-off points for the WHO recommendations (<400 grams/day or ≥400 grams/day). For descriptive purposes, characteristics of participants according to cognitive impairment were expressed as number and percentages or means and standard deviations (SD) and bivariate analyses performed to determine the association between characteristics and cognitive impairment. The T-Student test, Pearson chi-squared test and the exact Fisher test were used to compare means of continuous variables and proportions of categorical variables (Table 1). The association between cognitive impairment and each quartile of total fruit and vegetables intake (Model II) and WHO recommendation levels (Model III), was evaluated in two separate multivariate logistic models (Table2). For each category of co-variates, Wald test P values and their 95% CIs for the OR were calculated. All covariates with P values <0.05 or those reported in the biomedical literature as determinants of cognitive impairment such as APOE, were retained in the final models (Model II & III from Tables 2). Covariates which changed original estimated coefficients (OR) for more than 20% were also included as confounders. We explore the interaction between fruit and vegetable intake and years of education with all other covariates in the Models and the two significant interactions found were presented in Table 3 (for Model II), and Table 4 (for Model III). We excluded observations with missing values since analyses using LR test with observations with missing values could lead to spurious results [29]. Statistical analysis was performed using R statistical package v.3.0.1. (http://www.r-project.org/) and SPSS v.18.

**Table 1:** General characteristics of the study participants of the SPAH study according to cognitive impairment.

		Cognitive function ≤20.9	Cognitive function >20.9	
Category	Sub category	Result (%)	Result (%)	P-value
Age group (yrs)	65–69	28 (3.4)	792 (96.6)	<0.001(1)
	70–74	33 (6.5)	477 (93.5)	
	75–80	34 (11.4)	265 (88.6)	
	80 or more	52 (23.6)	168 (76.4)	
Sex	Women	107 (9.6)	1013 (90.4)	<0.001(2)
	Men	40 (5.5)	689 (94.5)	
Fruit and vegetables consumption (quartiles g/day)	0–176	60 (13.0)	403 (87.0)	<0.001(1)
	177–326	38 (8.2)	424 (91.8)	
	327–507	27 (5.8)	435 (94.2)	
	508–850	22 (4.8)	440 (95.2)	
Fruit and vegetables consumption (WHO) g/day	<400	117 (10.3)	1020 (89.7)	<0.001(2)
	≥400	30 (4.2)	682 (95.8)	
Education (yrs)	None	116 (16.5)	587 (83.5)	<0.001(1)
	1 to 3	31 (3.2)	950 (96.8)	
	4 or more	0 (0.0)	165 (100.0)	
Income per capita (Brazilian Real)	<240	87 (15.5)	474 (84.5)	<0.001(1)
	241–360	28 (7.8)	330 (92.2)	
	361–700	22 (4.7)	448 (95.3)	
	>700	10 (2.2)	450 (97.8)	
Physical Activity	Sedentary	39 (15.5)	161 (80.5)	<0.001(1)
	Moderately active	23 (11.7)	173 (88.3)	
	Highly active	85 (5.8)	1368 (94.2)	
Smoking	No	129 (8.1)	1467 (91.9)	0.35(2)
	Yes	18 (7.1)	235 (92.9)	
Alcohol consumption	No	79 (6.7)	1102 (93.3)	0.21(2)
	Yes	42 (7.9)	490 (92.1)	
Fish consumption	Never	79 (10.9)	647 (89.1)	<0.001(1)
	Some days	66(6.4)	972 (93.6)	
	Frequently	2 (2.4)	83 (97.6)	
Diabetes	No	102 (7.4)	1269 (92.6)	0.14(2)
	Yes	36 (9.3)	352 (90.7)	
Hypertension	No	31 (8.1)	353 (91.9)	0.49(2)
	Yes	113 (7.9)	1317 (92.1)	
APOE	Reference	97 (8.2)	1093 (91.8)	0.88(1)
	Protective	14 (8.8)	146 (91.3)	
	Low risk	24 (7.8)	282 (92.2)	
	High risk	1 (3.6)	27 (96.4)	
Previous stroke	No	115(6.8)	1583 (93.2)	<0.001(2)
	Yes	32 (21.2)	119 (78.8)	
BMI (Kg/m^2^)	<23	55 (11.0)	444 (89.0)	0.007(1)
	23–27.9	57 (7.7)	687 (92.3)	
	28–29.9	14 (7.0)	185 (93.0)	
	≥30	14 (4.4)	302 (95.6)	
HDL-cholesterol (mg/dl)	≤50	62 (9.2)	609 (90.8)	0.06(2)
	>50	76 (7.1)	998 (92.9)	

(1) Chi-Square test;

(2) Fischer test;

**Table 2:** General characteristics of the study participants of the SPAH study according to cognitive impairment.

		Cognitive function ≤20.9	Cognitive function >20.9	
	N	mean (sd)	mean (sd)	P-value(1)
Cognitive Function	1849	17.7 (3.5)	27.6 (2.8)	<0.001
Age in years	1849	77.5 (8.1)	71.5 (5.6)	<0.001
BMI (Kg/m^2^)	1771	24.7 (4.4)	26.0 (4.7)	<0.001
Glycaemia (mg/dl)	1743	109.6 (40.7)	111.4 (42.5)	0.63
Cholesterol (mg/dl)	1745	206.0 (48.1)	210.1 (43.7)	0.30
HDL-cholesterol(mg/dl)	1745	54.0 (13.8)	56.2 (14.3)	0.09
LDL-cholesterol (mg/dl)	1724	124.9 (36.8)	125.2 (34.9)	0.92
Triglycerides (mg/dl)	638	165.0 (135.0)	148.0 (93.2)	0.34
Systolic B.P. (mmHg)	1772	151.4 (29.0)	145.6 (25.6)	0.01
Diastolic B.P. (mmHg)	1772	85.9 (15.1)	86.3 (13.3)	0.78

(1) Student test.

**Table 3:** Associations between the prevalence of cognitive impairment and quartiles of fruit and vegetables intake and WHO recommendations for daily fruit and vegetables intake SPAH study^1^.

		Model I^2^		Model II^3^		Model III^4^	
		OR (95% CI )	P-value^5^	OR (95%CI)	P-value^5^	OR (95% CI)	P-value^5^
Age group	65–69	1		1		1	
	70–74	1.70 (0.93–3.11)	0.09	1.43 (0.76–2.68)	0.27	1.47 (0.78–2.77)	0.23
	75–80	3.46 (1.90–6.28)	<0.001	2.50 (1.33–4.72)	0.004	2.63 (1.39–4.99)	0.003
	80 or more	8.12 (4.61–14.30)	<0.001	5.02 (2.72–9.28)	<0.001	5.26 (2.83–9.77)	<0.001
Sex	Women	1		1		1	
	Men	0.53 (0.34–0.84)	<0.001	0.57 (0.34–0.95)	0.03	0.44 (0.25–0.78)	0.005
Fruit and vegetables consumption (quartiles in g/day)	0–176	1		1			
	177–326	0.58 (0.34–0.98)	0.04	0.68 (0.39–1.18)	0.17	–	–
	327–507	0.48 (0.28–0.84)	0.01	0.60 (0.33–1.11)	0.10	–	–
	508–850	0.38 (0.21–0.69)	0.001	0.63 (0.33–1.21)	0.16	–	–
Fruit and vegetables consumption (WHO) g/day	<400	1				1	
	>400	0.40 (0.25–0.65)	<0.001	–	–	0.53 (0.31–0.89)	0.02
Education	None	1		(a)(b)	–	(a)(b)	–
	1 or more years	0.23 (0.14–0.36)	<0.001				
Income per capita (Brazilian Real)	<240	1		1		1	
	241–360	0.46 (0.28–0.77)	0.003	0.60 (0.35–1.04)	0.07	0.61 (0.35–1.04)	0.07
	361–700	0.33 (0.19–0.58)	<0.001	0.47 (0.26–0.85)	0.01	0.47 (0.25–0.85)	0.01
	>700	0.13 (0.05–0.31)	<0.001	0.21 (0.08–0.52)	<0.001	0.22 (0.09–0.54)	<0.001
Physical Activity	Sedentary	1					
	ModeratelyActive	0.43 (0.21–0.89)	0.02	(a)	–	(a)	–
	Highly Active	0.33 (0.20–0.54)	<0.001				
Smoking	No	1					
	Yes	1.07 (0.56–2.03)	0.85	NS	–	NS	–
Alcohol consumption	No	1				1	
	Yes	2.11 (1.31–3.40)	0.002	NS	–	1.87 (1.12–3.13)	0.02
Fish consumption	Never	1					
	Some days/Frequently	0.55 (0.37–0.83)	0.004	NS	–	NS	–
Diabetes	No	1					
	Yes	1.24 (0.78–1.98)	0.37	NS	–	NS	–
Hypertension	No	1					
	Yes	0.74 (0.46–1.22)	0.24	NS	–	NS	–
APOE	Neutral	1		1		1	
	Protective	0.96 (0.49–1.89)	0.91	0.90 (0.44–1.84)	0.78	0.88 (0.43–1.80)	0.72
	Risk	0.89 (0.52–1.52)	0.67	1.02 (0.58–1.81)	0.95	1.06 (0.60–1.88)	0.84
Previous stroke	No	1		1		1	
	Yes	2.65 (1.54–4.55)	<0.001	2.23 (1.22–4.09)	0.009	2.25 (1.23–4.14)	0.009
BMI	<23	1					
	23–27.9	0.79 (0.49–1.25)	0.31	NS	–	NS	–
	28–29.9	0.61 (0.29–1.26)	0.18				
	≥30	0.51 (0.26–1.00)	0.05				
HDL-cholesterol	≤50 mg/dl	1		(b)	–	(b)	–
	>50 mg/dl	0.70 (0.45–1.07)	0.10				
Glycaemia (mg/dl)		1.00 (0.99–1.01)	0.44	NS	–	NS	–
Cholesterol (mg/dl)		1.00 (0.99–1.01)	0.68	NS	–	NS	–
LDL-cholesterol (mg/dl)		1.00 (0.99–1.01)	0.51	NS	–	NS	–
Triglycerides (mg/dl)		1.00 (0.99–1.01)	0.89	NS	–	NS	–
Systolic B.P. (mmHg)		1.01 (1.00–1.02)	0.02	NS	–	NS	–
Diatolic B.P. (mmHg)		1.00 (0.99–1.02)	0.27	NS	–	NS	–

1 Odds Ratio (OR) and 95% confidence interval (CI) obtained from logistic regression models.

2 Model I Adjusted for age and sex, 1503 valid cases.

3 Model II Adjusted for factors in Model I and fruit and vegetables consumption in quartiles, 1503 valid cases.

4 Model III Adjusted for factors in Model I and fruit and vegetables consumption according to daily WHO recommendations, 1503 valid cases.

5 Two tailed P values based on Wald tests.

NS: Non-Significant.

(a)(b) Significant interaction between education-physical activity and education-HDL-cholesterol respectively. Odds Ratio (OR) and 95% confidence interval (CI) presented in Tables 3 and 4.

**Table 4:** Interaction between years of education and physical activity, and years of education and HDL-cholesterol in Model II: (quartiles of fruit and vegetables intake), SPAH study^1^.

		Physical Activity			HDL-cholesterol	
		Sedentary	Moderately Active	Highly Active	≤50 mg/dl	>50 mg/dl
Education	None	1	0.77 (0.31–1.90)	0.75 (0.39–1.46)	1	1.25 (0.72–2.16)
	1yr or more	1	0.12 (0.03–0.57)	0.14 (0.06–0.31)	1	0.20 (0.09–0.46)

1 Odds Ratios (OR) and their 95% Confidence Interval (95% CI).

## Results

Of 2072 elderly individuals recruited, 1849 were included in the final analyses, 1120 women (60.6%) and 729 men (39.4%). Six participants were excluded due to missing data on fruit and vegetable consumption, 105 individuals were excluded because they were diagnosed with dementia at baseline, 10 individuals due to missing data on cognitive function assessment and 102 participants were excluded because they reported improbable consumptions of fruit and vegetables with intakes ranging from 850 g/d to 1325 g/d (≥3.5 standard deviations from the mean).

Table 1 and Table 2 show the general characteristics of the population classified according to cognitive impairment, scores ≤20.9, and without cognitive impairment scores above 20.9. An 8% (n = 147) of the participants presented cognitive impairment, (mean = 17.7, SD = 3.5) against 92% (n = 1702) without impairment (mean = 27.6, SD = 2.8). Those with cognitive impairment compared against those without cognitive impairment were significantly older (77.5 years *vs.*71.5 years p<0.001), there were more women than men (9.6% *vs*. 5.5% p<0.001), they reported significant lower daily combined intakes of fruit and vegetables (mean 272 grams/d vs. 364 grams/d), and very few achieved WHO recommendations (4.2% *vs.* 10.3% p<0.001). Among those without cognitive impairment compared with those with cognitive impairment, the majority had 1 or more years of education, had significantly higher monthly income per capita, were more physically active, reported a higher daily intake of fish, and reported less episodes of stroke. They also had lower average levels of systolic blood pressure and higher BMI.

The odds ratios (OR) and 95% confidence intervals (CI) for cognitive impairment obtained from logistic regression models are shown in Table 3 for three sets of models. Model I consists of each variable only adjusted by age and sex. Model II, is the final model for the association between cognitive impairment and quartiles of combined fruit and vegetable intake with further adjustments for co-variates. Model III is the final model for the association between cognitive impairment and daily WHO fruit and vegetables recommendations with further adjustments. Both models (II & III) included two significant interactions: one between cognitive impairment and ‘years of education and physical activity’ interaction term, and the other with ‘years of education and HDL-cholesterol level’ interaction term (Table 4 and Table 5, respectively).

**Table 5:** Interaction between years of education and physical activity, and years of education and HDL-cholesterol in Model III: (WHO recommendations), SPAH study^1^.

		Physical Activity			HDL-cholesterol	
		Sedentary	Moderately Active	Highly Active	≤50 mg/dl	>50 mg/dl
Education	None	1	0.78 (0.32–1.91)	0.78 (0.40–1.51)	1	1.23 (0.71–2.13)
	1yr or more	1	0.15 (0.03–0.66)	0.16 (0.07–0.37)	1	0.21 (0.09–0.48)

1 Odds Ratios (OR) and their 95% Confidence Interval (95% CI).

Thus, results from Model II show that no significant association was found between quartiles of fruit and vegetable intake and cognitive impairment but Model III shows that intakes from ≥400 grams/day were associated with a decreased prevalence of cognitive impairment OR = 0.53 95% CI (0.31–0.89). In both models, men and higher income were associated with a decreased prevalence of cognitive impairment, whereas having had a previous stroke episode was associated with an increased prevalence of cognitive impairment. In Model III usual alcohol consumption was also associated with increased prevalence of cognitive impairment.

Tables 4 and 5 show the OR and 95% CI for cognitive impairment for the interaction between each level of education and physical activity, and each level of education and HDL–cholesterol in Models II and III. Thus, illiterate participants in the physical categories moderately active and highly active compared to sedentary showed a mild protective effect against cognitive impairment which did not reach significance OR 0.77 95% CI (0.31–1.90) and OR 0.75 95% CI (0.39–1.46), respectively. However, for those with one or more years of education there was a strong modification effect in both categories of physical activity, for moderate activity OR 0.12 95% CI (0.03–0.57) and for highly active OR 0.14 95% CI (0.06–0.31) *versus* sedentary. In Model III we observed that this interaction was very similar thus, among those with one or more years of education for moderate and highly physically active categories *versus* sedentary there was a strong decreased prevalence of cognitive impairment OR 0.15 95% CI (0.03–0.66), and OR 0.16 95% CI (0.07–0.37), respectively.

A similar interaction was found between years of education and HDL-cholesterol. Therefore, among illiterate participants, those with HDL-cholesterol levels >50 mg/dl versus levels ≤50 mg/dl showed a mild increased association with cognitive impairment which did not reach significance 1.25 95% CI (0.72–2.16) and OR 1.23 95% CI (0.71–2.13) for Models II and III, respectively. However, among those with one or more years of education and HDL-cholesterol levels >50 mg/dl versus ≤50 mg/dl we observed a strong modification effect with a strong decrease in prevalence of cognitive impairment, OR 0.20 (0.09–0.46) and OR 0.21 (0.09–0.48) for Model II and III, respectively.

## Discussion

Although quartiles of fruit and vegetable intake were not associated to lower prevalence of cognitive impairment, WHO recommendations for daily intakes ≥400 grams/day were significantly associated with a 47% decreased prevalence of cognitive impairment. Moreover, an effect modification between years of education and physical activity and years of education and blood levels of HDL was found. Thus, within the group of those with 1 or more years of education compared to no education, to be physically active compared to sedentary or to have HDL-cholesterol levels above 50 mg/dl compared to levels ≤50 strongly decreased prevalence of cognitive impairment.

The present study is one of two Brazilian studies that assessed the association between cognitive decline and consumption of fruit and vegetables and the only one to evaluate this association using WHO recommendations. The other Brazilian cross-sectional study was conducted with 1558 individuals >60 years and showed that those with consumption of less than 5 portions per week of fruit and vegetables compared to those who consumed more than five portions per week had worse cognitive function using the Mini Mental State Examination test (MMSE), OR = 1.94 95% CI (1.46–2.59) [11].

A recent systematic review of cohort studies showed evidence that increased intake of vegetables is associated with lower risk of dementia and lower rates of cognitive imapirment, however, no evidence was found for the same association with fruit [7].

The results of the present research suggest a protective role for those meeting WHO recommendations against cognitive impairment despite that only a minority of our population 19.8% 95% CI (18.1–021.5) met WHO recommendations. In this study, the prevalence of those with intakes ≥400 g/day of fruit and vegetables is in concordance with findings from a WHO survey carried out in several developing countries, including Brazil where only a 22% of individuals >18 years old showed daily intakes ≥400 g/day of fruit and vegetables [30]. Furthermore, results from the World Health Survey data carried out in Brazil in 2003 noted that for participants >65 years of age the prevalence of fruit and vegetable intake, according to the WHO recommendations, was 20.6% for women and 14.8% for men, and this intake was associated with higher income and higher education [31]. Most recently, the Brazilian Household Budget Survey conducted between 2008–2009, estimated that less than 10% of the population spent enough of their family budget on fruit and vegetables to be able to attain WHO recommendations [32]. In a recent reanalyses of these data it was observed that the intake tended to increase linearly with increasing level of household income [33].

Most population studies suggest that the consumption of fruit and vegetables worldwide is below the current WHO recommendations, and the consumption of individuals meeting recommendations is associated with socioeconomic factors. The consumption of fruit and vegetables in the elderly included in the SPAH study proved to be clearly inadequate. Even in this relatively homogeneous population in relation to socioconomic characteristics, inadequate fruit and vegetable intake was associated with adverse socioeconomic conditions (lower education level and lower per capita income). It is known that lower levels of education and income are associated with higher risk of developing non-communicable diseases, including cognitive decline and dementia [34]. Several studies have shown that higher educational levels are associated with lower risk of developing dementia [35].

In fact, previous results from our population showed that illiteracy, poor occupational achievement and low income accounted for 22.0% to 38.5% of the cases of dementia and that low educational attainment earlier in life generally triggers a cascade of social adversities that increased the risk of dementia later in life [36].

Thus, a recent Brazilian post mortem coss-sectional study showed that even a few years of formal education were associated with less cognitive impairment independent of neuropathlogic burden [37].

Our study found an important interaction, which was not anticipated *a priori,* between years of education and physical activity, and between years of education and HDL-cholesterol levels and decreased prevalence of cognitive impairment, confirming the importance of social determinants in healthier choices and better quality of life in this elderly population. Even though our population presented very low levels of years of education, those with 1 or more years showed a strong decrease of prevalence of cognitive impairment. Recent evidence demonstrates that literacy and the first years of education are associated with remarkable changes in cortical network organization and function [38]. On the other hand, physical activity has also been implicated as an important component not only of improved cardiorespiratory fitness, but its benefits would extend also to improved cognitive function and reduced risk for dementia [14], [39], [40]. In a cohort study in China with 27,651 people aged between 50 and 85, those who were classified as physically active had a 28% decreased risk of having mild cognitive impairment, and this relationship was dose-dependent OR = 0.72 95% CI (0.58–0.89) [15].

In the U.S. Nurses’ Health Cohort Study, women who had higher intakes of fruit and vegetables had higher schooling levels and were more physically active [41]. In a Dutch cohort study with 2613 participants, those individuals with higher intakes of fruit and vegetables (highest quintile) had reduced cognitive decline, higher educational level, fewer risk factors (such as smoking, alcohol intake and sedentary lifestyle) and higher levels of HDL-cholesterol [2]. The association between education and levels of HDL-cholesterol may be mediated by the best dietary patterns and also by increased participation in physical activity among people with higher education levels.

The SPAH study had a high response rate (91.4%) and this contributed to minimize potential selection bias. The performance of the interviews and assessments in the households made less likely that the non-participation of the elderly has been due to comorbidities, also avoiding selection bias, which in this case would be related to participation of healthier older. Attempts were also made to minimize interviewer bias by masking observers to disease status and the objectives under study. In this study only 1.6% (n = 29) of the interviews were answered by the informants. Analysis was repeated excluding these and results were similar to those presented here, so we decided to include them in our analyses. We also attempted to minimize the influence of unadjusted confounding by including in the Models all the variables that we found to be associated with cognitive impairment or those reported in the scientific literature including APOE gene.

As with any epidemiological study that aims to classify participants according to their cognitive functioning, misclassification could have occurred [20], [42]. However, the protocol used included applying CSI-D which was developed to provide equal performance in the assessment of cognitive functioning of individuals from different cultures and educational levels. The CSI-D was translated into Portuguese and validated for the elderly population, with robust sensitivity (94%) and specificity (94%) to detect cognitive impairment in individuals with low education [20]. As with any tool for data collection, the CSI-D has a degree of non-differential classification that is inherent to the instrument, since it is based on memory. The resulting error in measuring the cognitive impairment from CSI-D would be expected to lead to a dilution of the magnitude of the association.

The assessment of physical activity was not performed using a standardized questionnaire and consisted merely of self-reported question straightforward. However, our results are in line with other studies that found an association using more thorough tools [14], [39], [40]. We believe that the use of a low sensitivity tool for the assessment of physical activity, did not bias the relation found in the present study.

The SPAH included a population living in low-income areas of São Paulo city. The demographic and socioeconomic characteristics of the study population were very similar to those aged 65 or over in the Brazilian census of 2000 and 2010 supporting the external validity of our results [18], [43].

We believe that, with caution, the associations observed in this study can be generalized to the elderly population of Brazilian urban centers and possibly to the elderly in other low- and middle-income countries. Although the cross-sectional nature of the SPAH study does not permit the establishment of a causal relationship, this study suggests that the results examined here play an important role in the cognitive function of this socially deprived population.

## Conclusions

In this disavantaged population those who attained WHO recommendations for daily intakes of fruit and vegetables had a significantly decreased prevalence of cognitive impairment. Moreover, those with even 1 or more years of education compared with those with none, or the fact of being physically active as opposed to sedentary had an important impact on the prevalence of cognitive impairment.

It is expected that the incidence of cognitive impairment in elderly Brazilians increases concomitantly with the rapidly aging population. Through research, we need not only to have a more comprenhensive understanding of the biological processes which lead to cognitive impairment, but also to have a broader view of the social determinants of this health problem. Research must not only focus on increasing life expectancy but also on improving quality of life. The present results show that promoting education and health policies to encourage and enable healthy lifestyles in disadvantanged populations could make a significant difference. The development of strategies to prevent or delay age associated cognitive impairment should be a priority for public health, in order to achieve the best health possible when older.
