# Correction: Education and WHO recommendations for fruit and vegetable intake are associated with better cognitive function in a disadvantaged Brazilian elderly population: a population-based cross-sectional study

**DOI:** 10.1371/journal.pone.0104139

**Published:** 2014-07-25

**Authors:** The PLOS ONE Staff

There is an error in the funding statement for this article. Please refer to the correct funding statement below:

This study was funded by Wellcome Trust, U.K. (GR066133MA); MS and PRM were partly funded by CNPq-Brazil. Maria Pastor-Valero was a visiting lecturer at the Preventive Medicine Department, Faculty of Medicine, São Paulo University funded by Ministerio da Educação, Brasil, programa CAPES/professor visitante do exterior, processo BEX 13827/12-0. The funders had no role in study design, data collection and analysis, decision to publish, or preparation of the manuscript.

In addition, there is an error in the co-author Simon Almeida da Silva’s first name. The correct name for this co-author is Simone Almeida da Silva.
